# Incremental Validity and Informant Effect from a Multi-Method Perspective: Assessing Relations between Parental Acceptance and Children’s Behavioral Problems

**DOI:** 10.3389/fpsyg.2016.00664

**Published:** 2016-05-10

**Authors:** Eva Izquierdo-Sotorrío, Francisco P. Holgado-Tello, Miguel Á. Carrasco

**Affiliations:** ^1^Department of Personality, Assessment and Psychological Treatments, Faculty of Psychology, National University of Distance EducationMadrid, Spain; ^2^Department of Behavioral Science Methodology, Faculty of Psychology, National University of Distance EducationMadrid, Spain

**Keywords:** incremental validity, multiple informants, parental acceptance-rejection, behavioral problems, children, hierarchical regression, structural equations models, informant effect

## Abstract

This study examines the relationships between perceived parental acceptance and children’s behavioral problems (externalizing and internalizing) from a multi-informant perspective. Using mothers, fathers, and children as sources of information, we explore the informant effect and incremental validity. The sample was composed of 681 participants (227 children, 227 fathers, and 227 mothers). Children’s (40% boys) ages ranged from 9 to 17 years (*M* = 12.52, *SD* = 1.81). Parents and children completed both the Parental Acceptance Rejection/Control Questionnaire (PARQ/Control) and the check list of the Achenbach System of Empirically Based Assessment (ASEBA). Statistical analyses were based on the correlated uniqueness multitrait-multimethod matrix (model MTMM) by structural equations and different hierarchical regression analyses. Results showed a significant informant effect and a different incremental validity related to which combination of sources was considered. A multi-informant perspective rather than a single one increased the predictive value. Our results suggest that mother–father or child–father combinations seem to be the best way to optimize the multi-informant method in order to predict children’s behavioral problems based on perceived parental acceptance.

## Introduction

The progress of psychology is inextricably linked to the development of new and more refined methods and strategies for measuring psychological concepts, models, and intervention programs (Eid and Diener, 2006). A multi-informant approach offers insights into scientific phenomena and can contribute to confirming psychological theories in a way that a single-informant approach cannot. Due to the complexity of constructs evaluated and developmental factors that take place in children’s psychological adjustment, their assessment is mainly multimodal (e.g., rating scales, interviews, and observations), multi-informant (e.g., child, parents, teachers, and mates), and/or multi-trait (Eyde et al., 1993; Ollendick and Hersen, 1993; Mash and Terdal, 1997; Duhig et al., 2000; Meyer et al., 2001; Johnston and Murray, 2003; Achenbach, 2006; Hunsley and Mash, 2007). Specifically for informant assessment, the most reliable source of information on a target’s psychological characteristics is not to be found in his or her self-ratings, nor it is guaranteed by single informant ratings; rather, it is found in the combination of the judgments from the community of the target’s knowledgeable informants. According to this, the multi-informant assessment is mostly accepted by the psychological assessment community as an adequate and useful procedure, since rarely is a unique measure sufficient for providing all the required information needed to form an accurate judgment (Meyer and Archer, 2001; Garb, 2003; De Los Reyes and Kazdin, 2004; Carrasco et al., 2008; Hughes and Gullone, 2010). However, informant effects represent bias that can derive from the use of the same source of information in the assessment of different traits, the knowledge of informants, the observability of assessed traits, the judgment of informants, or the social desirability, among other factors (Cheng and Furnham, 2004; Neyer, 2006). For these reasons, determining the extent to which an informant effect is affecting the assessment of constructs and its relations is an important goal in determining the real construct validity. Individual reports often yield inconsistent data and discrepancies that can create considerable uncertainties in designing interventions and drawing conclusions from research (Klein, 1991; Epkins, 1993; Jané et al., 2000; De Los Reyes and Kazdin, 2004, 2005, 2006; Achenbach, 2006; Goodman et al., 2010; De Los Reyes et al., 2015). For instance, associations between constructs tend to be largest: (*a*) when a single informant is used, because of shared method variance (Neyer, 2006); (*b*), when the assessment of interventions has a large effect on parent reports vs. observed child behaviors of children’s externalizing problems (Tarver et al., 2014); or (*c*) when family members experience their interaction differently and therefore have dissimilar views on parenting and parent child relations (e.g., Lanz et al., 2001; Hoeve et al., 2009). A key reason for these uncertainties originates from the near-exclusive focus on mental health research as applied to whether informant discrepancies reflect measurement error or reporting biases (e.g., Richters, 1992; De Los Reyes, 2011). Consequently, what remains unclear is whether a multi-informant approach to assessment validly captures contextual variations displayed in children’s behavioral problems or whether it instead reflects different perceptions or beliefs about what a symptom is, and, finally, which informants ought to be included in assessments of children and adolescents.

Regarding this last point, another important issue from a multi-informant approach is the differential contribution of a particular source of information in relation to others. That is, the incremental validity or degree to which adding a new informant to the assessment consistently increases the predictive power and decision making (Garb, 2003; Hunsley, 2003; Hunsley and Mash, 2005). Unfortunately, the incremental validity inherent in using and combining multiple assessment methods has not undergone wide empirical testing in the literature on either adult or child assessment (Mash and Terdal, 1997; Hunsley, 2002). Thus, strong psychometric properties of the individual measures are necessary but do not provide sufficient conditions to ensure the incremental validity of incorporating these measures into the assessment process. Furthermore, not only is the research that deals directly with incremental validity in child assessment relatively small, the incremental validity of mothers’ vs. fathers’ reports has seldom been tested (Johnston and Murray, 2003).

With regard to cross-informant use, some studies support the incremental value of adults’ over children’s information when externalizing problems are measured (Loeber et al., 1991; Carrasco et al., 2008). However, the use of adults’ information in children’s assessment does not always augment the value of using only one source of information (Biederman et al., 1990). On the other hand, for older children, when assessing internalizing problems or covert behaviors, there is some evidence for the incremental value of youth self-reports over parents reports (Langhinrichsen et al., 1990; Cantwell et al., 1997; Johnston and Murray, 2003).

One of the most consistent observations in the field of child assessment is the correspondence levels between informants’ reports, which range from low to moderate in magnitude (Achenbach et al., 1987; Duhig et al., 2000; Achenbach, 2011; Markon et al., 2011; De Los Reyes et al., 2015). The evidence usually shows that pairs of informants who observed children in the same context (e.g., pairs of parents or pairs of teachers) tend to show greater levels of correspondence than pairs of informants who observed children in different contexts (e.g., parent and teacher). Accordingly, some studies have found that the cross-informant agreement was moderate to high between mother and father, and moderate to low between father–child and mother–child pairs (Grigorenko et al., 2010; Weitkamp et al., 2013). Correspondence between mothers and children tend to be higher than correspondence between fathers and children (Grigorenko et al., 2010) and mother–child reports tend to find a greater endorsement than father–child reports (Lapouse and Monk, 1958; Achenbach et al., 1987; Stanger and Lewis, 1993; De Los Reyes et al., 2015). Also, the confluence of informants’ reports about children’s externalizing problems (e.g., aggression and hyperactivity concerns) tends to be higher than that concerning internalizing problems (e.g., anxiety and depression). In this regard, maternal and paternal reports show moderate correspondence when rating internalizing behavior problems in children and a larger correspondence in ratings of externalizing behavior problems in children (Achenbach et al., 1987; Duhig et al., 2000; Grigorenko et al., 2010). This evidence may reflect the greater correspondence between reports of directly observable behaviors than internalized behaviors. There is also evidence supporting claims that the degree of acquaintance between parents and children is a factor that leads to different parental ratings (Hughes and Gullone, 2010). The variability of correspondence found between the different pairs of informants is probably reflective of both the potential informant effect and the differential contribution of each source of information to the assessment’s target. Furthermore, we would like to remark that the variation of the responses will be due to real differences from individual subjects, and the variation of the subjects on the variable won’t be a continuous uniform distribution, but its favorable or unfavorable position on the studied object will be according to their perception (Likert, 1932).

This study tries to explore from a multi-informant approach the relations between parental acceptance and children’s internalizing and externalizing problems. Perceived parental acceptance is one of the main factors involved in children’s psychological adjustment, as is shown from the interpersonal acceptance-rejection theory (IPARTheory; Rohner, 1986; Rohner et al., 2012). Parental rejection (the opposite of parental acceptance) implies the absence or a significant withdrawal of parental warmth, affection, care, comfort, concern, nurturance, support, or love, and the presence of a variety of physically and psychologically hurtful behaviors and effects (Rohner and Khaleque, 2005; Rohner et al., 2012). Meta-analysis studies on this subject have found that rejection has consistently negative effects on the psychological adjustment and behavioral functioning of both children and adults worldwide (Khaleque and Rohner, 2002; Rohner and Khaleque, 2005; Rohner et al., 2012). The same body of research also shows that children who perceive their parents as being rejecting tend to experience distress, and in turn develop a specific cluster of internalizing (i.e., emotional instability, depression) and externalizing (i.e., aggression, delinquency) problems (McLeod et al., 2007; Hoeve et al., 2009; Rohner and Khaleque, 2010; Khaleque and Rohner, 2012; Khaleque, 2015; Ramírez-Lucas et al., 2015). However, no studies from this perspective have been conducted, to our knowledge, that explore either the informant effect or the incremental validity of parents’ and children’s perceived parental acceptance on externalizing and internalizing behavioral problems. Accordingly, no specific results are expected and no particular hypotheses are going to be tested. The first aim of this study is to test for evidence of informant effects related to the links between parental acceptance and children’s behavioral problems as measured by children, fathers, and mothers through a round-robin design, in which all informants rate all targets. The second aim is to explore the incremental validity of the informants. Specifically, we deal with two questions: (1) Are there significant informant effects predicting children’s behavioral problems based on perceived parental acceptance? (2) What is the incremental validity of the children’s perceived parental acceptance over the parent’s perceived parental acceptance in predicting the children’s behavioral problems?

## Materials and Methods

### Sample

The sample was composed of 681 participants (227 children, 227 fathers, and 227 mothers). Children’s (40% boys; *n* = 90) ages ranged from 9 to 17 years (*M* = 12.52, *SD* = 1.81): 37% (*n* = 61) were between 9 and11 years, 47% (*n* = 107) were between 12 and 13 years, 20% (*n* = 46) were between 14 and 15, and 6% (*n* = 13) were between 16 and 17 years.

All of the children attended school, the majority lived in two-parent households (91%), and the mean number of siblings was three. Of the parents, 88% of fathers and 70% of mothers were employed. Occupational titles for mothers and fathers (respectively) were: major professionals (17 and 17%), lesser professionals (40 and 33%), semi-skilled workers (18 and 26%), and unskilled workers (25 and 24%). The mothers’ and fathers’ education levels were: university studies (40 and 35%), high school studies (40 and 57%), and primary studies (20 and 8%).

This sample is part of a larger sample of a general study about parental acceptance and children’s psychological adjustment in the Spanish population. Children were selected according to mother–father–child matched participation. This sample represents 22% of the total sample (*N* = 1036). The total sample was randomly selected from public schools and publically funded private schools in different cities and communities of Spain. The participation rate of the total families was 91.5%.

No significant differences were found between participant and non-participant families in the demographic variables (i.e., child’s sex, age, and socioeconomic level).

### Measures

All measures were filled in by children, mothers, and fathers using the appropriate versions of the instruments described below.

#### Parental Acceptance

Four versions of the *Parental Acceptance-Rejection/Control Questionnaire* were used to report on perceived parental acceptance, two for children (mother and father versions, one to report about each parent) and two for parents (one version for mothers and another version for fathers). Children filled in both mother and father versions (*Parental Acceptance-Rejection/Control Questionnaire*, Child PARQ/Control: *mother-short version for children* and Child PARQ/Control: *father -short version for children).* Mothers filled in mother versions and fathers filled in father versions (*Parental Acceptance-Rejection/Control Questionnaire*, PARQ/Control: Mother*- short version for parents* and, PARQ/Control: father*- short version for parents;* Rohner, 1990; Rohner and Khaleque, 2005; Spanish adaptation by Del Barrio et al., 2014). The short versions of the PARQ/Control for children and for parents consist of 29-item. The PARQ/Control for children is a self-reporting questionnaires with four scales measuring warmth/affection [e.g., “My mother (father) says nice things about me”], hostility/aggression [e.g., “My mother (father) gets angry at me easily”], indifference/neglect [e.g., “My mother (father) pays no attention to me”], and undifferentiated rejection [e.g., “My mother (father) does not really love me”], plus a parental control (permissive-strictness) scale built into it. The PARQ/Control for mothers and fathers are self-reports with the same scales as the version for children; the difference with the children version is that items ask about the mother or father her/himself (e.g., “I get angry at my son easily”). The mother and father versions of the PARQ/Control (short forms) are identical, with the exception of the title changing according to which parent is being assessed. In all versions items are scored on a 4-point Likert-type scale ranging from 4 (*almost always true*) through 1 (*almost never true*). The sum of the first four scales (24 items) constitutes a measure of overall perceived maternal and paternal acceptance/rejection (with the entire warmth scale reverse scored). A greater score indicates a perception of greater parental rejection. Evidence regarding the validity and reliability of the PARQ/Control has been very well supported (Khaleque and Rohner, 2002; Rohner and Khaleque, 2005). Coefficient alphas for the total score in this sample are 0.88 for fathers and 0.97 for mothers in the children versions; and 0.88 for fathers and 0.88 for mothers in the parent version.

#### Children’s Behavioral Problems

Two versions from the Achenbach System Evidence Based Assessment (Achenbach and Rescorla, 2007) were used to report on the children’s behavioral problems: one for children (YSR) and one for parents (CBCL). Fathers and mothers inform separately about the children’s behavioral problems on the CBCL version. The *Youth Self-Report* (YSR) is composed of two parts, the first assessing various psychosocial skills and competences, and the second consisting of a check-list of 112 items assessing a large number of behavioral problems, which are aggregated into two broad dimensions: internalizing (anxiety/depression, withdrawal, somatic complaints) and externalizing (breaking rules, aggressive behavior) problems. The items are scored on a 3-point Likert-type scale with anchors of 0 (*not true*), 1 (*somewhat or sometimes true*), and 2 (*very true or often true*). The *Children’s Behavioral Check List* (CBCL) is similar to YSR, with the exception of having one item more (113 “Other problems”). For the purpose of this study, we only use the check-lists and the two broad dimensions: externalizing and internalizing behavioral problems.

For this sample, the Cronbach’s alphas were 0.75 for the internalizing scale, and 0.73 for the externalizing scale on the YSR version; 0.79 and 0.78 for the internalizing scale, and 0.80 and 0.77 for the externalizing scale on the father-CBCL and mother-CBCL, respectively.

### Procedure

Once the cluster sample of schools was selected, an authorization from the school board and an informed consent form from each child’s responsible guardian were collected. Participation was voluntary. The instruments were administered collectively to each school class group in their own classrooms by research personnel trained for this task.

To explore the potential informant effect, we started with the correlated uniqueness model MTMM (Multitrait-multimethod Matrix; Byrne, 1998). According to the correlated uniqueness model, if the different sources are adding systematic variability to the model, we should find significant correlations between errors of the dependent variables reported by the same informant. At the same time, no matter what the global fit of the model is, a significant increase in the model fit should be noted. Second, we used a different hierarchical regression analysis to determine the magnitude of the incremental validity.

Data was analyzed using LISREL 8.9 and SPSS version 20.0 for Windows (SPSS WIN).

### Design and Variables

A round-robin design was employed, in which fathers, mothers, and children separately completed all the instruments used. The independent variables were parental acceptance levels as perceived by children, mothers, and fathers. The dependent variables were children’s externalizing and internalizing problems, reported separately by fathers, mothers, and children.

### Results

In **Table 1** is included the correlation matrix among the variables used. According to the Multitrait-Multimethod matrix logit, if any informant effect exists the Monosource-Multitrait correlation should be higher than the Multisource-Multitrait one. If we focus on the dependent variables, we observe that the correlation between the internalizing and the externalizing problems informed by children (*r*_int-ext_) is 0.54 (monosource-multitrait). This value is higher than other multisource-multitrait correlations such as *r*_int-pext_ = 0.15; *r*_int-mext_ = 0.19; *r*_pint-pext_ = 0.12; or *r*_mint-ext_ = 0.02. These results should take us to think about a possible informant effect. The same pattern is found in other variables. Thus, the correlation intra-informant for the same two variables is higher than the correlation inter-informants.

**Table 1:** Correlation matrix.

	PARQP	PARQM	EXT	INT	MPARQ	MEXT	MINT	PPARQ	PEXT	PINT
PARQP	–									
PARQM	0.56**	–								
EXT	0.40**	0.41**	–							
INT	0.23**	0.17*	0.54**	–						
MPARQ	0.30**	0.39**	0.23**	0.08	–					
MEXT	0.30**	0.23**	0.34**	0.19**	0.34**	–				
MINT	0.10	0.09	0.02	0.17*	0.19**	0.36**	–			
PPARQ	0.38**	0.24**	0.23**	0.14*	0.38**	0.27**	0.17*	–		
PEXT	0.30**	0.20**	0.29**	0.15*	0.34**	0.75**	0.27**	0.27**	–	
PINT	0.20**	0.14*	0.12	0.13*	0.19**	0.39**	0.48**	0.18**	0.58**	–
**Mean**	35.48	33.00	13.49	17.33	36.30	5.12	6.73	36.91	4.69	5.45
***SD***	8.71	8.96	9.70	10.38	4.79	4.85	8.09	6.18	4.54	5.07

Ext. Prob., Externalizing problems; Int. Prob., Internalizing problems; Pac, paternal acceptance; Mac, maternal acceptance. *p < 0.05, **p < 0.01.

**FIGURE 1:**
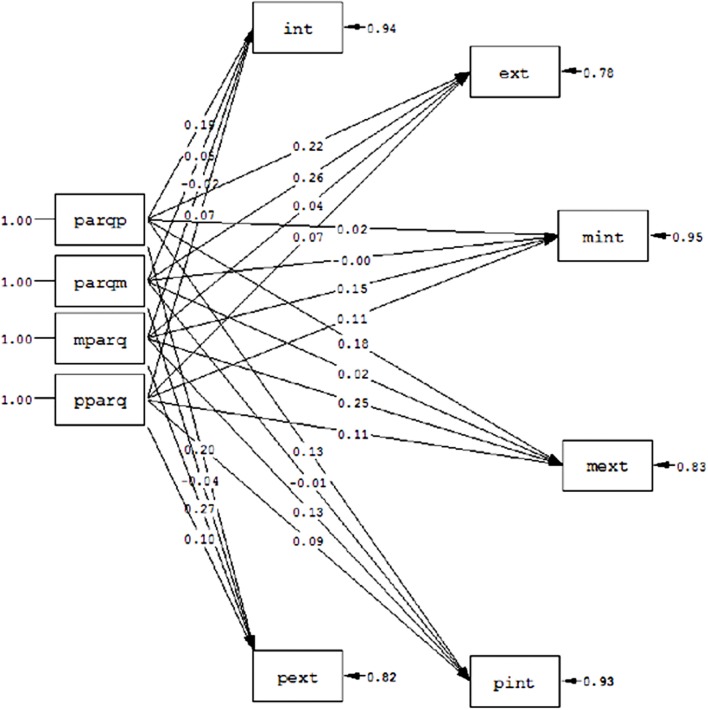
**Parental acceptance predicting children’s behavioral problems from a multi-informant method with uncorrelated errors (Model 1).** Parqp, paternal acceptance reported by children; parqm, maternal acceptance reported by children; mparq, maternal acceptance reported by mothers; pparq, paternal acceptance reported by fathers; int, internalizing problems reported by children; ext, externalizing problems reported by children; mint, internalizing problems reported by mothers; mext, externalizing problems reported by mothers; pext, externalizing problems reported by fathers; pint, internalizing problems reported by fathers.

**FIGURE 2:**
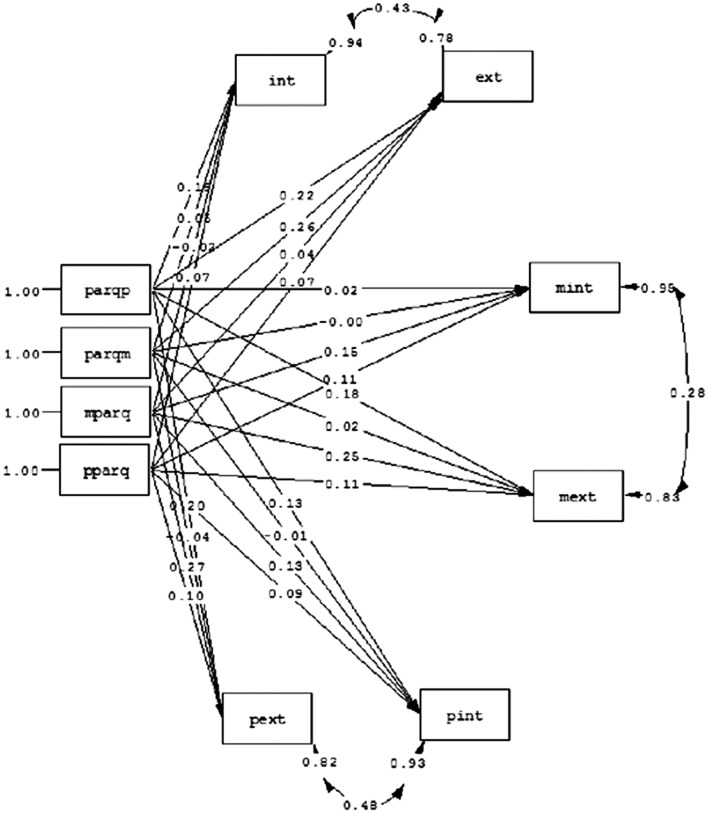
**Parental acceptance predicting children’s behavioral problems from a multi-informant method with correlated errors (Model 2).** Parqp, paternal acceptance reported by children; parqm, maternal acceptance reported by children; mparq, maternal acceptance reported by mothers; pparq, paternal acceptance reported by fathers; int, internalizing problems reported by children; ext, externalizing problems reported by children; mint, internalizing problems reported by mothers; mext, externalizing problems reported by mothers; pext, externalizing problems reported by fathers; pint, internalizing problems reported by fathers.

In order to obtain more evidences about the informant effect, we tested two models. In the first one (model 1), all the PARQ measures (PARQP, PARQM, MPARQ, and PPARQ) were predictors of all the criterion variables (INT, EXT, MINT, MEXT, PINT, and PEXT; **Figure 1**). The second model (model 2), was essentially the same, but included the correlations between the errors of each criterion variable reported by each informant (children, mothers, and fathers; **Figure 2**). We established that if we observed significant correlations between these errors in the second model, and the fit was improved, then it could be reasonable to think about an informant effect.

The fit indexes obtained for the first model were: χ^2^ = 482.66, df = 21; *p* = 0.00; CFI = 0.57; RMSEA = 0.30; GFI = 0.35; AGFI = 0.35; GFI = 0.75; RMR = 0.15. For model 2, we obtained: χ^2^ = 236.01, df = 18; *p* = 0.00; CFI = 0.79; RMSEA = 0.22; GFI = 0.86; AGFI = 0.56; RMR = 0.12.

Logically, in terms of fit indexes, both models are not necessarily accepted because we are not looking for a predictive model to explain the relationship between the variables. According to our premise, we should test whether the errors of the various criterion measures from the same informant are correlated. In this sense, model 2 improves the fit of the model 1 (Δχ^2^ = 246.65; Δdf = 3), and the correlations between the errors of the criterion variables reported by the same source of information are significant [*e*_int_ext_ = 0.43, *Critical Ratio* (CR) = 9.74; *e*_mint_mext_ = 0.28, CR = 5.31; *e*_pint_pext_ = 0.43, CR = 10.69].

These results show a significant effect of the informant. As we can see in **Figure 2**, children and fathers are the informants that add more variability to the model; that is, the covariance of errors between children’s internalizing and externalizing problems are higher when they are reported by fathers and by children than when they are reported by mothers. In order to quantify the magnitude of the contributions of the various informants, and their incremental validity, we conducted six hierarchical regression analyses.

The results from the hierarchical regression analyses are shown in **Table 2**. The contribution of perceived parental acceptance on behavioral problems is organized by the three informants (mothers, fathers, and children) and by the children’s externalizing and internalizing problems.

**Table 2:** Hierarchical regression analyses predicting children’s behavioral problems by multi-informants.

	Mother informant				Father informant				Child informant			
	Externalizing problems		Internalizing problems		Externalizing problems		Internalizing problems		Externalizing problems		Internalizing problems	
	β	*R^2^/*Δ*R^2^*	β	*R*^2^/Δ*R*^2^	β	*R*^2^/Δ*R*^2^	β	*R*^2^/Δ*R*^2^	β	*R*^2^/Δ*R*^2^	β	*R*^2^/Δ*R*^2^
**Step 1** Age Sex	-0.04 0.13	*R*^2^ = 0.02 Δ*R*^2^ = 0.02	-0.07 0.05	*R*^2^ = 0.00 Δ*R*^2^ = 0.00	-0.05 0.13*	*R*^2^ = 0.02 Δ*R*^2^ = 0.02	-0.01 0.11	*R*^2^ = 0.01 Δ*R*^2^ = 0.01	-0.13* 0.24**	*R*^2^ = 0.08 Δ*R*^2^ = 0.08**	0.05 0.16*	*R*^2^ = 0.03 Δ*R*^2^ = 0.03*
**Step 2** Pac by Father	0.20**	*R*^2^ = 0.06 Δ*R*^2^ = 0.04**	0.13	*R*^2^ = 0.02 Δ*R*^2^ = 0.01	0.24**	*R*^2^ = 0.08 Δ*R*^2^ = 0.06**	0.13	*R*^2^ = 0.03 Δ*R*^2^ = 0.01	0.17**	*R*^2^ = 0.10 Δ*R*^2^ = 0.02**	0.13*	*R*^2^ = 0.05 Δ*R*^2^ = 0.02*
**Step 3** Mac by Mother	0.29**	*R*^2^ = 0.13 Δ*R*^2^ = 0.07**	0.15*	*R*^2^ = 0.04 Δ*R*^2^ = 0.02*	0.29**	*R*^2^ = 0.15 Δ*R*^2^ = 0.07**	0.18*	*R*^2^ = 0.05 Δ*R*^2^ = 0.02*	0.13	*R*^2^ = 0.12 Δ*R*^2^ = 0.02*	0.00	*R*^2^ = 0.05 Δ*R*^2^ = 0.00
**Step 4** Pac by child	0.14	*R*^2^ = 0.15 Δ*R*^2^ = 0.02	0.02	*R*^2^ = 0.04 Δ*R*^2^ = 0.00	0.19*	*R*^2^ = 0.17 ΔR^2^ = 0.02*	0.14	*R*^2^ = 0.07 Δ*R*^2^ = 0.01	0.17*	*R*^2^ = 0.01 Δ*R*^2^ = 0.13**	0.16*	*R*^2^ = 0.09 Δ*R*^2^ = 0.04**
Mac by child	0.03		0.00		0.04		0.00		0.27**		0.08

Pac, paternal acceptance; Mac, maternal acceptance. *p < 0.05, **p < 0.01.

When the informant referencing the child’s behavioral problems is the mother, the maternal acceptance reported by mothers shows the largest increment of *R*^2^, especially for externalizing problems. However, paternal acceptance reported by fathers made a significant contribution to externalizing problems (not internalizing), and maternal acceptance reported by mothers made a significant contribution to both internalizing and externalizing behavioral problems. Parental acceptance (maternal or paternal) perceived by children does not make any significant contribution to behavioral problems. Parental acceptance reported by fathers and maternal acceptance reported by mothers considered together become to explain 19% of the variance on externalizing problems.

When the informant referencing the child’s behavioral problems is the father, the same pattern was found, with the exception of the instance of externalizing problems seen in step 4, wherein children make a significant contribution. Parental acceptance reported by fathers, mothers, and children considered together become to explain the 40% of the variance on externalizing problems.

Finally, when the informant referencing the child’s behavioral problems is the child, the largest increase occurs in step 4, when children report on parental acceptance. Nevertheless, both paternal and maternal acceptances were significant predictors of externalizing problems (not internalizing problems), while only paternal acceptance was significant for internalizing problems. The increase in the variance explained by the parental acceptance perceived by children is 13% for externalizing problems and 4% for internalizing. Parental acceptance reported by fathers and children (the significant sources of information) considered together become to explain the 11% of the variance on externalizing problems and 14% on internalizing problems.

## Discussion

Method effects and incremental validity are two important issues for construct validity. The analysis of empirical similarities and differences between self and others as informants contribute to the knowledge of consistency of measures, its reliability and accuracy, and its validity in terms of behavior prediction (Kenny, 1994; Neyer, 2006). This study dealt with two questions: (1) Are there significant informant effects predicting children’s behavioral problems from perceived parental acceptance? (2) What is the incremental validity of children’s perceived parental acceptance over parents’ perceived parental acceptance in predicting children’s behavioral problems?

In relation to the first question, our findings confirm a significant informant effect, which shows that the predictive values are different from one informant to the others when predicting behavioral problems in children based on perceived parental acceptance. Consequently, the magnitude of relations in terms of behavior prediction between parental acceptance and children’s externalizing and internalizing problems depends on the source of information used (i.e., children, mothers, or fathers). When the informant speaking on the child’s behavioral problems is the mother, maternal acceptance perceived by mothers and paternal acceptance perceived by fathers are the best predictors of children’s externalizing problems, while the best predictor for internalizing problems is only the maternal acceptance informed by mothers. The information provided by children about parental acceptance does not make any contribution to the behavioral problems reported upon by mothers. Likewise, the same pattern emerges when the informant about the child’s behavioral problems is the father, except that children make a significant contribution to informing on externalizing problems (not internalizing). However, when children act as informants on their own behavioral problems, the pattern found is completely different; maternal acceptance as assessed by mothers does not make any contribution to the children’s behavioral problems. Only paternal acceptance reported by fathers or children predicts the externalizing and internalizing problems; additionally, maternal acceptance reported by children predicts internalizing (not externalizing) problems.

The significant predictive value of perceived parental acceptance and children’s psychological adjustment is very well supported in family research (Khaleque and Rohner, 2012; Rohner et al., 2012), but no studies have been conducted to explore the informant effect of parental acceptance on children’s behavioral problems. Our results support this significant relation regardless of the source of information. Furthermore, our findings are consistent with previous studies that have found an informant effect reflected on the low or moderate confluence between children and parents on the information given by each of them (Achenbach et al., 1987; Rescorla et al., 2013; De Los Reyes et al., 2015). There are numerous prospective reasons for these results, such as the potential biased perception of informants (i.e., parents tending to perceive and inform about less or more problems than children), the information that informants use to rate the scales (i.e., family and school), conceptions of what constitutes abnormal behavior (Richters, 1992), the informants’ own emotional state (Chilcoat and Breslau, 1997; Najman et al., 2000; Berg-Nielsen et al., 2003), the closeness of parent–child relationships (Hughes and Gullone, 2010), or the observability of behaviors (De Los Reyes and Kazdin, 2005).

According to previous studies (Stanger et al., 1992; Duhig et al., 2000), our results support the different predictive utility that a multiaxial assessment approach may have in children’s outcomes, specifically in predicting the children’s externalizing and internalizing behavioral problems from the parental acceptance construct. In this regard, when parents report about the children’s behavioral problems, both fathers (paternal acceptance) and mothers (maternal acceptance) tend to be the best informants to predict externalizing problems, while mothers (maternal acceptance) excel at predicting internalizing ones. However, when children report about their own behavioral problems, children (paternal acceptance to externalizing and internalizing problems, and maternal acceptance to internalizing ones) and fathers (paternal acceptance) tend to be the best informants to predict all kinds of children’s behavioral problems.

Research does not yet allow us to make a conclusion about to what extent maternal or paternal acceptance will make a higher or lower contribution to children’s psychological problems. Some studies suggest that maternal parenting is more strongly associated with children’s emotional and behavioral problems than paternal parenting (Rosnati et al., 2007; Meunier et al., 2012), while other studies find that the opposite is true (Flouri and Buchanan, 2002; Khaleque and Rohner, 2011). Probably on the basis of this contribution differences could be the externalized–internalized nature of behavioral problems, as well as the informant effect. Accordingly, the greater contribution of maternal acceptance to the children’s problems could be explained by the closeness of the mother–child relationship and by the fact that mothers tend to have more knowledge about the children’s behavioral problems (mainly about the internalizing ones), possibly because mothers generally spend more time with their children than fathers (Renk et al., 2003; De Los Reyes and Kazdin, 2005), or because mothers could be perceived by their offspring to have higher interpersonal power and prestige than fathers (Carrasco et al., 2014). Paternal acceptance may become more relevant to externalizing problems than internalizing because of the nature of father–child relationships, which tend to be more focused on leisure activities (Torres et al., 2014) and goal-oriented behaviors (Leaper et al., 1998; Tenenbaum and Leaper, 2003). The informant effect that our study shows is consistent with the studies that found a higher contribution of paternal acceptance vs. maternal acceptance when the informants are children (Flouri and Buchanan, 2002; Bosco et al., 2003; Khaleque and Rohner, 2011) or teachers (Mattanah, 2001). Maternal parenting tends to be a stronger predictor of children’s behavioral problems when parents are the source of information (Gryczkowski et al., 2010), but this is not always confirmed (Hoeve et al., 2009).

Regarding the second question concerning how incremental validity was also affected by the source of information on the children’s behavioral problems, our results suggest that there are differential contributions of one source of information over the others and a subsequent incremental validity related to which combination of sources is considered. More specifically, when the informant about the child’s behavioral problems is the mother, both father’s and mother’s information about parental acceptance increases the predictive validity for externalizing problems, but only the mother’s information does this (maternal acceptance) for internalizing. However, when the informant about the child’s behavioral problems is the father, then mothers, fathers, and children increase the predictive validity for externalizing problems. Nevertheless, only the mother’s information about maternal acceptance has significant predictive value on internalizing problems. Finally, when the informant about the child’s behavioral problems is the child, then mothers, children, and fathers increase the predictive validity for externalizing problems, but only fathers (not mothers) and children do this for internalizing problems. It is important to highlight that mothers have the higher incremental validity when parents (mothers or fathers) inform about children’s problems, but that children make the larger contribution to incremental validity when they self-report about their own behavioral problems. These results support the children’s ability to be introspective and to assess their own thoughts and feelings even better than adults (Bidaut-Russell et al., 1995; Johnston and Murray, 2003). These results are also consistent with the studies that support the incremental value of adult informants compared with the child’s reports on externalizing problems (Loeber et al., 1990, 1991).

Furthermore, our results support that single informants (parents or children) produced significantly stronger effects than multiple informants (parents and children). That is, when the same informant provides information about parental acceptance (predictor) and the children’s outcomes (dependent variable), this single informant tends to reach the higher incremental validity. It is probably due to shared method variance (Campbell and Fiske, 1959). This effect may be particularly prominent when children are the source of information. Although asking children to report on parenting and their own behavioral problems can lead to inflated effect size estimates, children could provide the best information about themselves and the perceived parent–child relationships. The higher incremental validity of mothers on children’s internalizing problems is consistent with the higher predictive value of maternal acceptance on internalizing behaviors, as previously discussed.

When fathers are the source of information, the rest of the informants (children and mothers) add significant incremental validity. This could be because fathers sometimes have less knowledge of children’s day-to-day lives, meaning that more information is needed from mothers and children to predict children’s behavioral problems. However, when children are the source of information, the incremental validity is mainly added by fathers. This may be because of overlapped information from mothers and children, as these would share more information about the emotional lives of the children. It is consistent with the higher agreement between mothers and children than between fathers and children (Schneewind and Ruppert, 2013; Leung and Shek, 2014). The closer relationship of mother and child can account for a higher concurrence on the information provided by these informants, and therefore, the parent with a closer relationship will give much redundant information when added to the one given by the child. In cultures like that of Spain, where gender and parental roles are still quite differentiated, it is common for mothers to spend more time than fathers with the children, which could be a reason why the mother does not add significant information when the child is used as the primary informant. Similarly, when the mother is the primary informant, the child does not add additional significant information.

Considering all these results as a whole, it can be concluded that the child is the best source of information about parental acceptance when we are trying to predict the children’s behavioral problems (both externalizing and internalizing) reported by the own child. However, when the behavioral problems are informed by the parents, the parental acceptance information provided by them will be the data with better predictive value for children’s externalizing problems. This changes when we deal with children’s internalizing problems that are reported by the parents, in which case the mother’s information will be the most predictive one.

A few limitations should be considered for future lines of research. First, this study focused on the general population instead of a clinical sample, meaning that generalization of the current findings to clinical populations should be made with caution, and future research should consider how these two samples may differ both quantitatively and qualitatively. Second, the lack of analysis by sex and age as moderators may be particularly relevant (Crick and Grotpeter, 1995; Johnston and Murray, 2003; Hughes and Gullone, 2010) in terms of informant effect and incremental validity. Studies about sex and age differences in the perception of parental acceptance and the expression of internalizing or externalizing problems symptoms may lead to variations in informant agreement and in relationships between parental acceptance and children’s symptoms. Third, the parent’s social desirability could minimize their reports about any adverse parenting experiences (i.e., rejection) affecting the level of parent–child agreement. Four, different methods of evaluation such as observations, rating scales, and self-reports should be explored in addition to the informant method. Future studies conducted from a developmental and gender perspective with a multi-measure perspective and using clinical samples are advised in order to bring more light to the informant effect and incremental validity.

Despite the above limitations, the findings of the present study have important practical implications. Considering previous analysis, a multi-informant perspective rather than a single should be considered in order to increase the predictive value and the incremental validity when we try to predict children’s internalizing and externalizing problems. Our results suggest that mother–father or child–father informant pairs seem to be the way to optimize the combinations of sources of information in order to predict children’s behavioral problems from parental acceptance. Nevertheless, a child may give enough information to make future decisions, and if we have to add only one informant to the assessment, this should be the father. There is a clear need for more research from a multi-method perspective in the child assessment field, rather than having blind faith in a “more are better” approach to getting informants (Johnston and Murray, 2003), which will lead to an optimization of empirically based children’s assessment (Carrasco et al., 2008).

## Author Contributions

The tasks of each individual author are described in the folloing lines. EI-S: Bibliographic review, preparation of data matrices, drafting the theoretical contents, drafting the discusion, writing and preparing manuscript for sending. FH-T: Collection of data, statistical analysis, drafting the methodology and results. MC: Collection of data, statistical analysis, drafting the methodology and results, theoretical contents review, team coordination.

## Conflict of Interest Statement

The authors declare that the research was conducted in the absence of any commercial or financial relationships that could be construed as a potential conflict of interest.
